# Notes and News

**Published:** 1895-02-02

**Authors:** 


### Notes and News.

Collected and Communicated.

A grave oversight appears to have crept into the newly-constructed vessels of the United States Navy. It appears that in no less than ten ships no accommodation for the sick is provided.

The once-neglected subject of sick-room cookery is now making universal progress. At the Royal Infirmary, Edinburgh, its teaching is not now confined to the nursing staff, but demonstrations are held for the instruction of the medical students.

It is difficult to understand how the new Board of Management of the Chelsea Hospital for Women, having themselves invited and obtained the assistance of a committee consisting of Sir William Broadbent, Sir William Priestley, Sir Walter Foster, and Mr. Henry Morris, can have dared to treat their recommendations with contempt as they have done. Surely the troubles which have beset the Chelsea Hospital for Women during recent months are grave enough to warrant the governors in believing that the gentlemen who constitute the present Board of Management would show more statesmanship and appreciation of public opinion than to thus re-open the whole controversy by ignoring the report of the medical experts and re-electing the old medical staff without change. It is not to be wondered at that Sir William Broadbent and Sir William Priestley have declined to accept the office of consulting physicians to the hospital, and that Mr. Jonathan Hutchinson should have resigned the office of consulting surgeon in order to mark their sense of the conduct of the Committee of Management. An immediate explanation is demanded in the public interest from the management of the Chelsea Hospital for Women, who seem to us to have needlessly imperilled the very existence of this institution for any useful purpose by electing the staff in a manner which is as unjustifiable as it is opposed to the best interests of the institution and all connected with it. In the circumstances we should not be surprised to hear that Lord Cadogan had definitely declined to resume office as president of the institution.

An International Congress on Childhood is announced to be held at Florence during the spring of the present year.

The subject of a universal language for the use of medical men is again occupying attention. Opinion shows far too great a divergance to raise expectation as to the adoption of the idea.

A new hospital has just been opened at Bangor by the Mayoress. It has been erected by the corporation for the use of the borough. A former Mayor of Bangor, Mr. Charles Pierce, gave £500 towards its equipment.

We are glad to observe, in perusing the reports of the hospitals throughout the country, that the uniform system of accounts is increasingly adopted. Amongst the latest to progress in this direction are the West Sussex, East Hants and Chichester Infirmary, and the Kilmarnock Infirmary.

A scheme to raise £35,000, for the purpose of rebuilding the Royal South Hants Infirmary at Southampton, is now before the public. Whilst an average of 85 beds are occupied out of the 120 existing at the present infirmary, it is felt that a larger and improved building is really needed to adequately provide for the sick poor of Southampton. The neighbourhood has not supported the institution sufficiently in the past, but that is no reason why fresh efforts should not alter this in the future.

An order of physicians in America, called "Sundown Doctors," has just come to an end. It had long been the practice of Government clerks in the Pension Office to practice medicine out of office hours. The system was naturally much objected to by regular medical practitioners. Only recently has a sufficiently weighty reason been found to warrant Government prohibition. It has been established that contagion has been carried into Government departments by means of these "doctors," who henceforward are forbidden to follow the calling of medicine.

A function not easily forgotten was the dinner given last week in Dr. Jameson's honour by his old friends and former fellow-students of University Hospital. It was essentially a friendly entertainment, and, owing to the absence of reporters, it had somewhat the air of a big private party of unusually large dimensions. Many were the pleasant greetings interchanged by men who had come from far and near to meet the famous traveller. Of course, Dr. Jameson's own speech was the great feature of the evening, and it was one of peculiar interest.

It seems strange that in a country so dominated with aristocratic prejudices as Austria that the medical profession should receive such far higher social distinction than is awarded to them in England. Dr. Edward Albert, Professor of Clinical Surgery, and Dr. Hermann Freiherr von Widerhofer, Professor of Children's Diseases at Vienna, have been made members of the Austrian House of Lords. Dr. von Widerhofer, of course, was already a member of the aristocracy by birth, but this applies to no other doctor so distinguished in Austria.

